# Microvascular Decompression for a Patient with a Glossopharyngeal Neuralgia: A Technical Note

**DOI:** 10.7759/cureus.1494

**Published:** 2017-07-20

**Authors:** Edgar Gerardo Ordónez-Rubiano, Cristian C García-Chingaté, Saney Rodríguez-Vargas, Hernando A Cifuentes-Lobelo, Tito A Perilla-Cepeda

**Affiliations:** 1 Neurosurgery Department, Fundación Universitaria De Ciencias De La Salud, Hospital de San Jose/Hospital Infantil Universitario de San José

**Keywords:** neuralgia, microvascular decompression, glossopharyngeal nerve diseases, glossopharyngeal nerve, cranial nerve injuries, neurosurgery

## Abstract

The glossopharyngeal neuralgia (GPN) constitutes approximately 0.2-1.3% of all facial pain syndromes. The GPN is a syndrome of neuropathic pain characterized by paroxysmal pain episodes localized in the posterior tongue, tonsil, throat, or external ear canal. The first-line treatment is pharmacological. Patients who are refractory to medical therapy can be treated surgically with microvascular decompression (MVD) or sectioning the IX nerve and the upper rootlets of the X nerve. We aim to describe the technical nuances of MVD of the IX cranial nerve with a targeted inferior mini-craniotomy in a patient with a neurovascular compression.

## Introduction

Glossopharyngeal neuralgia (GPN) (a.k.a. vagoglossopharyngeal neuralgia) is a rare condition, with an estimated incidence of 0.8 cases per 100,000 persons per year [1], with predilection among females, principally within the fifth decade of life [2], and corresponds to 0.2-1.3% of all facial pain syndromes [3]. GPN is a syndrome of neuropathic pain characterized by paroxysmal pain episodes localized in the posterior tongue, tonsil, throat, and/or the external ear canal. Vagal involvement can produce bradycardia, syncope or asystole. Common triggers include eating, swallowing and speaking [4]. GPN occurs more frequently on the left side and involvement is bilateral (not simultaneously) in only 2% of the cases [5]. The diagnosis of GPN is primarily clinical, and complimentary imaging can be performed, including computed tomography (CT) or magnetic resonance imaging (MRI) scans, which can reveal adjacent tumors, neurovascular conflicts, arteriovenous malformations (AVMs), demyelinating lesions, or an elongated styloid process involving the IX and X cranial nerves (CN). The first-line treatment is pharmacological, and can be initiated with anticonvulsant medication including carbamazepine, gabapentin, oxcarbazepine, or pregabalin. If the pain is refractory, the treatment can be surgical, including rhizotomy of CNs IX and X, or with microvascular decompression (MVD) if neurovascular conflicts are detected. We aim to describe the surgical technique of MVD with a targeted lateral inferior suboccipital mini-craniectomy in a patient with a GPN. We also attempt to review the technical nuances of this treatment option.

## Case presentation

A 57-year-old male presented to the emergency department with an 11-year right pharyngeal pain that increased in severity. It was a burning pain, with a score on the pain visual analogue scale 10 out of 10, irradiated to the right base of the tongue and to the distal pharynx, approximately with 30 seconds in duration per crisis, which was exacerbated by physical activity, swallowing, and with the elevation of the voice volume. Prior failed medication with pregabalin 600 mg/day, carbamazepine 600 mg/day and topic lidocaine was recorded. The patient developed a new crisis the day before the consult and had no other symptoms associated to the crisis. No relevant clinical antecedents were found. On examination, the following vital signs were recorded: (1) blood pressure of 116/76 mm Hg, (2) pulse of 80 beats per minute, (3) respiratory rate of 16 breaths per minute, and (4) oxygen saturation of 97%. The neurological examination demonstrated a right pharyngeal hyperalgesia, but no other positive findings were noted. No any active infectious process was detected as well. The rest of the physical exam was unremarkable.

**Figure 1:**
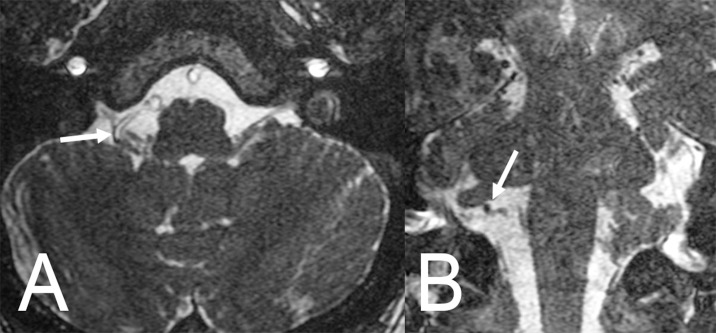
Non-enhanced brain magnetic resonance imaging (MRI). T2 high-resolution (FIESTA) axial (A) and coronal (B) slices showing the vessel involved in the neurovascular compression syndrome in the meatus of the right jugular foramen between the right IX cranial nerve and the posterior and inferior cerebellar artery (arrows).

**Figure 2:**
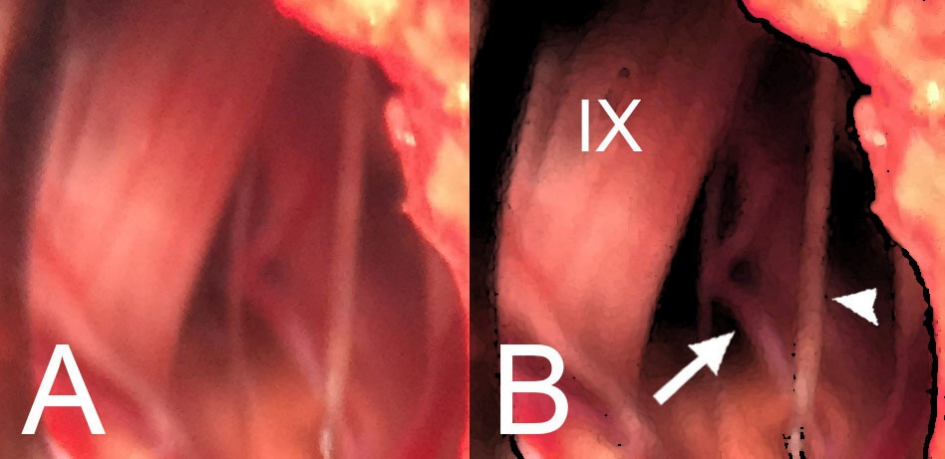
Intraoperative images before decompression. (A) An infra- and latero-floccular microsurgical approach on the right side is shown. The offending vessel, a perforator artery coming from the right posterior inferior cerebellar artery, ventral to the root entry zone of the IX and X cranial nerves is shown. A previous dissection and freeing of the rootlets from the posterior inferior cerebellar artery perforator was performed. (B) A schematic illustration of the intraoperative microscopic view is denoting the right IX cranial nerve (IX), a perforator artery from the right posterior inferior cerebellar artery (arrow), and a rootlet of the right X cranial nerve (arrowhead).

**Figure 3:**
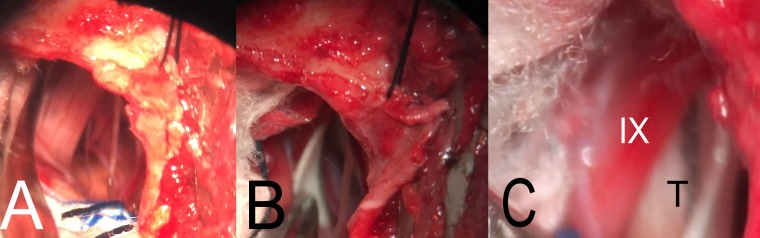
Intraoperative images of microvascular decompression of the glossopharyngeal nerve. (A) A right suboccipital retrosigmoid mini craniotomy was performed. A far lateral approach was achieved. The right IX cranial nerve, a perforator artery from the right posterior inferior cerebellar artery, and a rootlet of the right X cranial nerve were exposed. (B) The Teflon was placed separating the vessels from the IX and X cranial nerves. (C) The Teflon (T) was placed in the anterior and lateral aspect of the IX cranial nerve (IX), decompressing the nerve from the perforator artery from the right posterior inferior cerebellar artery (covered by the Teflon).

A right symptomatic GPN refractory to medical treatment was considered and an urgent enhanced MRI of the brain was performed. A neurovascular conflict between the right glossopharyngeal (IX) CN and the posterior inferior cerebellar artery (PICA) was observed on the glossopharyngeal meatus of the right jugular foramen, showing a IX CN compression in the axial and coronal slices of the 3D T2 high-resolution MRI (FIESTA) (Figure 1). The surgical decision-making group selected a right MVD as the best approach for this case (Figures 2-3).

The operating room (OR) course was unremarkable, as well as the following days during his hospital stay. A complete relief of the pain was successfully achieved. Comfort measures were provided. Two days after the procedure the patient was discharged for post-op follow-up in the outpatient clinic. After one year of follow-up, the patient presented no any recurrent pain.

## Discussion

The GPN is a rare entity. A little information is published regarding its worldwide distribution, however in 1981 Rushton, et al. showed a case series with 217 patients, describing their clinical features and management, finding a complete pain relief in 110 surgical patients [2]. In 2002, Patel, et al. presented their experience with more than 200 patients that underwent MVD, showing that 66.8% were females, with a mean age of 50.2 years, and a mean pain duration of 5.7 years [6]. The GPN can be classified by the clinical features, including the classic GPN (episodic pain) and the symptomatic GPN (continuous pain), and the second is classified by the etiology, including an idiopathic origin or a secondary nature (e.g., tumor, neurovascular compression) [7]. In this case, the pain was constant and refractory. No any trauma, infection or AVM was detected, classifying this case as a secondary symptomatic GPN [8]. There are three types of neurovascular compression (NVC): type I – NVC at the root entry zone (REZ) of the IX CN within the retro-olivary sulcus; type II – the vertebral artery causes NVC at the IX CN REZ by the shoulder of the artery, and the type III - a “sandwich-like” compression where the vertebral artery and the PICA perform a combination of NVC [8]. Depending on the type of compression the treatment varies. Different procedures can be performed, including gamma knife surgery (GKS), MVD or central procedures as tractotomy or trigeminal or caudal nucleus nucleotomy [3]. Our patient underwent MVD due to the neurovascular conflict observed in the MRI.

Surgical nuances

GPN tends to be refractory to pharmacological treatment, leading patients to elevated doses of different medication, thus exposing them to adverse reactions, making surgery a new possible treatment [3, 5]. Historically, the first surgical methods for GPN were based in extracranial ablation of the IX CN, proposed by Sicard and Robineau in 1920, however with important morbidity and high rate of recurrence due to lack of supraganglionar ablation. Posteriorly, Jannetta in the 1960s popularized the trigeminal tractotomy and the MVD of the glossopharyngeal nerve, with more acceptance of the rhizotomy, and lately the percutaneous procedures like the radiofrequency glossopharyngeal rhizotomy and GKS for GPN were popularized as well [5, 9]. Among the most important and relevant intracranial surgical procedures, there is the MVD, which shows symptomatic relief in 84.7% of cases, long-term pain relief in 64% of the cases, recurrence rates of 7%, and permanent complications in up to 5.5% [4, 9]. The procedure was first described primarily by Jannetta, who described the MVD for GPN step by step: once the anesthetic induction and intubation have been performed, the patient should be positioned in lateral decubitus fashion, fixing the head with a Mayfield head clamp, followed by the placement of an axillary roll. The neck should be narrowed with slight flexion and rotated approximately 10 degrees to the affected side. The vertex is tilted 15 degrees toward the floor. The shoulder is pulled out of the way and finally the patient is accommodated in such a way that the table can be rotated laterally or adjusted for a Trendelenburg position or reverse Trendelenburg position. For the incision, the mastoid eminence is initially demarcated, then a line is drawn from the external auditory canal to the inion to mark the transverse sinus. Then, a 3-4 cm arcuate or linear incision is performed, with the concave side toward the ear. Half of the incision should be above the mastoid notch or even more posteriorly in large, muscular or dolichocephalic patients. Subsequently, a retractor is placed and the bone is opened with a perforator, making sure to use bone wax in case of bleeding and filling the mastoid cells.

**Figure 4:**
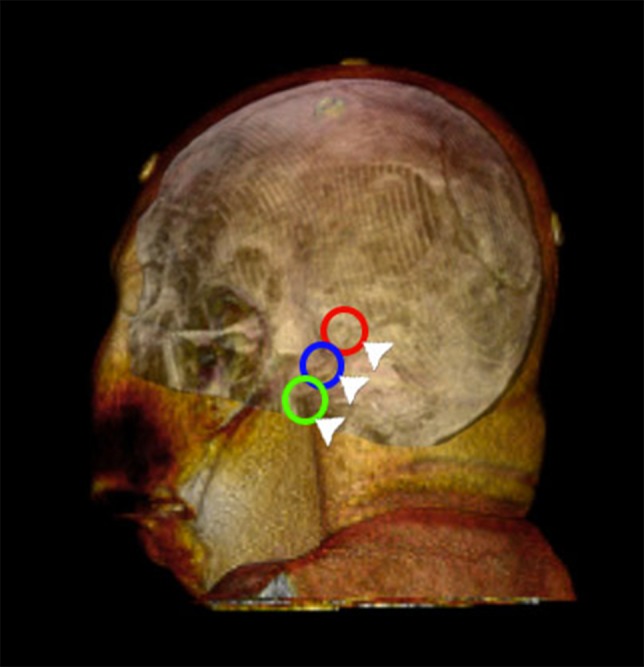
Targeted mini craniotomies for retromastoid-retrosigmoid approaches. (Red) The superior asterional minicraniotomy for CN V (mini extreme-lateral or microasterional), (blue) the middle for CNs VII and VIII, and (green) the inferior for the CNs IX to XII (mini far lateral). CN: Cranial nerve.

We propose to target the opening of the bone depending on the CN affected. Three different approaches could be performed. The superior for the V CN (mini extreme-lateral or microasterional), the middle for VII and VIII CNs (usual for the cerebellopontine angle), and the inferior for the IX to XII CNs (mini far-lateral) (Figure 4). Once the dura is exposed, it is incised and stretched. The form in which the dura is opened includes the L or reverse L shape, 3-5 mm parallel to the sigmoid sinus and to the floor of the posterior fossa, after which they are secured with sutures for a wider exposure. A retractor is placed under the cerebellum and raised from its inferolateral margin, after which the microscope is introduced, and the retractor is advanced anteriorly until the spinal part of the XI CN is observed, the arachnoid is dissected, which allows to elevate the cerebellum and expose the remaining CNs within the jugular foramen. Once the rootlets of the IX CN are identified, they are separated from the rootlets of the X and XI CNs. The involved vessel is identified and dissected before the decompression and finally, the Teflon is placed between the two structures.

If there is no NVC, the glossopharyngeal nerve and the upper bundle of the X CN can be sectioned [5]. Subsequently, a Valsalva maneuver is performed several times before closing. If there is venous bleeding, 1 mm of hydrogen peroxide can be applied to the area and then irrigated with saline solution. Once hemostasis is secured, the retractors and the cotonoids are removed and irrigated, preferably with a bulb rather than a syringe, because of their increased volume and flow of fluid at low pressure. The dura is closed with continuous stitches of nylon suture 4-0, the edge of the bone is again sealed with bone wax and finally, the wound is closed by planes in the usual way. The patient is transferred to the room or to the intensive care unit in case of complications.

Other techniques have been also described recently, derived from the original MVD described by Janneta, including microasterional and transcondylar approaches [9]. Risks of MVD include irritative cough, foreign body sensation in the throat, transient hoarseness or dysphagia, or persistent neurological damage with dysphagia and vocal cord paralysis in 8-19% of patients [4], which can be reduced by monitoring the X CN with electrodes in the false vocal cords or with placement of electrodes on the surface of the endotracheal tube to distinguish the sensory from the motor rootlets [5]. Mortality rates reported by authors such as Janneta are approximately 0.1%, however, there are reports of up to 5.8% [9].

The intracranial rhizotomy of the IX CN with or without vagal rhizotomy increases the risk of permanent vagal dysfunction by three times [9] and should be considered for patients who are not candidates for surgical management with MVD or in cases where the surgeon does not have enough experience with MVD [10]. On the other hand, the trigeminal tractotomy/nucleotomy is a procedure in which the descending trigeminal tract is destroyed in the medulla at the lower olive level via a posterior fossa craniectomy guided by CT, which can be used for cases of atypical pain, including GPN, with rates of improvement of almost 100% and no reports of complications; however, the series reported in the literature are small [10].

GKS and percutaneous radiofrequency thermocoagulation

GKS is performed in the glossopharyngeal meatus of the jugular foramen, with doses up to 80-90 Gy. GKS can be done before or after surgical management with MVD or rhizotomy or in cases where the patient refuses surgery. The efficacy of GKS for complete pain relief is up to 87%. Some risks of GKS include radiation of adjacent structures such as other CNs or other brainstem structures [4]. On the other hand, the percutaneous radiofrequency thermocoagulation is the selective destruction of A delta and C fibers. It can be performed through a single puncture in the posterior aspect of the styloid process, guided by CT. A sensory stimulation is with a 10 cm-needle with 0.5 V (50 Hz) to reproduce the pain of neuralgia, and with 1.0 V (2 Hz) to rule out the presence of motor fibers. Eventually, the patient is sedated and the radiofrequency thermal coagulation is performed at 70-80ºC for 120-180 seconds. The possible complications include the damage of vascular structures, such as the carotid artery, or the internal jugular vein, severe dysesthesias of the tongue, dysphagia and decreased gag reflex, making it recommended for patients whose condition is secondary to oropharyngeal cancer, or those who do not tolerate intracranial procedures.

## Conclusions

This paper illustrates a patient with a right GPN successfully treated with MVD, describing the technical nuances of MVD. We also propose a targeted minicraniotomy approach to NVC depending on the CN affected. MVD is one of the surgical procedures with the highest rates of pain relief for GPN secondary to vascular compression.
